# Exploring hot topics and evolutionary paths in the Diagnosis-Related Groups (DRGs) field: a comparative study using LDA modeling

**DOI:** 10.1186/s12913-024-11209-3

**Published:** 2024-06-21

**Authors:** Xinrui Chen, Meng Zhang, Qingqing Bu, Bo Tan, Peng Peng, Yilin Zhou, Yuqin Tang, Xiaoqin Tian, Dan Deng

**Affiliations:** School of Public Health, Chongqing Medical University, Chongqing, China

**Keywords:** Diagnosis-related group, LDA model, Hot topics, Evolutionary paths

## Abstract

**Background:**

This study reviews the research status of Diagnosis-related groups (DRGs) payment system in China and globally by analyzing topical issues in this field and exploring the evolutionary trends of DRGs in different developmental stages.

**Methods:**

Abstracts of relevant literature in the field of DRGs were extracted from the China National Knowledge Infrastructure (CNKI) database and the Web of Science (WoS) core database and used as text data. A probabilistic distribution-based Latent Dirichlet Allocation (LDA) topic model was applied to mine the text topics. Topical issues were determined by topic intensity, and the cosine similarity of the topics in adjacent stages was calculated to analyze the topic evolution trend.

**Results:**

A total of 6,758 English articles and 3,321 Chinese articles were included. Foreign research on DRGs focuses on grouping optimization, implementation effects, and influencing factors, whereas research topics in China focus on grouping and payment mechanism establishment, medical cost change evaluation, medical quality control, and performance management reform exploration.

**Conclusions:**

Currently, the field of DRGs in China is developing rapidly and attracting deepening research. However, the implementation depth of research in China remains insufficient compared with the in-depth research conducted abroad.

## Background

Owing to an aging population, medical innovation, and rising health needs, countries face increasing medical demands and rapidly growing service costs. Controlling medical expenses, conserving health resources, and providing affordable, high-quality medical care have become urgent problems for public hospitals [1]. To solve this practical problem, in the 1970s, diagnosis-related groups (DRGs) were established in the USA [2]. DRGs prioritize clinical similarity and consider resource consumption, grouping cases by disease severity, diagnosis complexity, treatment mode, and resource consumption to set payment standards [3]. As a refined medical payment tool, its appearance is conducive to protecting the rights and interests of patients, hospitals, and medical insurance. It can effectively meet the requirements of health expenditure control, hospital management, and medical resource allocation [4]. Therefore, different countries have developed and implemented adapted versions based on the payment method of DRGs in the USA and have achieved results in controlling medical expenses [5–7]. Since the late 1980s, China has introduced DRGs to control medical expenses and reduce patient burdens [8]. After more than 30 years of localization development and pilot programs, DRGs have proven effective in managing insurance fees, resource use, and medical quality [9, 10].

As medical insurance payment system reforms deepen, scholars worldwide are exploring diverse topics, generating new ideas, and proposing innovative theories and methods. DRGs in China started relatively late. Therefore, the overall quality of the papers published is lower compared to those from developed countries. Problems such as limited research scope, insufficient method innovation, and considerable homogenization persist [11]. Therefore, exploring the development status and research prospects of DRGs in China based on foreign research experiences is crucial. To this end, numerous researchers have extensively reviewed the literature on DRGs based on bibliometric tools such as CiteSpace and VOSviewer. They have also summarized the research directions in DRGs worldwide through keyword clustering [12, 13]. However, this method is limited to keywords, authors, and research institutions and cannot visually analyze the overall content of the text. Moreover, for documents with unclear semantic relations and a rough logical structure, keyword clustering can easily ignore the main keywords [14]. Latent Dirichlet Allocation (LDA) effectively addresses this problem by leveraging text information to enhance semantic keyword associations within mined topics [15]. However, few studies have applied the LDA topic model to the literature analysis of DRGs. Therefore, this study comprehensively reviews the Chinese and international literature on the DRG payment system since its implementation. Using the LDA topic model, it identifies hot topics and developmental trends, drawing on international experiences to support DRG implementation in China.

## Methods

### Source of data

The data sources for this study included the Web of Science (WoS) and China National Knowledge Infrastructure (CNKI). The WoS database encompasses diverse multidisciplinary, high-impact, international, and comprehensive academic journals. It ensures the representativeness and authority of the literature sources [16]. Considering its highest applicability to the LDA topic model, it was used as the database for this study [17]. CNKI is the largest and most comprehensive Chinese literature database, covering more than 99% of Chinese academic and practical journals. It ensures the representativeness and authority of the literature sources [18]. Both databases serve as effective tools for bibliometric analysis. Therefore, this study selected the WoS and CNKI databases as the data sources for English and Chinese literature, respectively. The following search strategies were employed: Topic = (“Diagnosis Related Groups”) OR Topic = (“Diagnosis Related Group”) OR Topic = (“Diagnosis-Related Groups”) OR Topic = (“Disease Related Group”) OR Topic = (“casemix”) OR Topic = (“case-mix”), Document Types = Article; Language = English, Indexes = Web of Science Core Collection (WoSCC) for English literature. Non-research literature and review articles such as conference notices, news reports, industry trends, policy documents, literature with incomplete data, and literature with unavailable full texts were excluded. Literature related to other professional terms with the same abbreviation “DRG,” such as Dorsal Root Ganglion, Digital Raster Graphics, and Dynamic Route Guidance System, was excluded. The cut-off date for data retrieval was April 12, 2023. A total of 6,758 and 3,321 English and Chinese articles, respectively, were screened.

The literature screening was independently conducted by two reviewers, who retrieved a literature record based on inclusion and exclusion criteria. Disagreements were resolved through consensus, and in cases of persistent differences, a third reviewer was consulted to assess and make a final decision. Furthermore, data collection from each screened literature record was independently conducted by two reviewers. A third reviewer then assessed the completeness and accuracy of the extracted data.

### Research method

We retrieved relevant literature on DRGs from the WoS Core and CNKI databases. We used the abstracts of the literature as text data. We preprocessed the text data by incorporating keyword information and removing stop words. We applied the LDA topic model to explore textual topics, identify hot topics in the field based on topic strength, and analyze the evolutionary trends of topics by calculating the cosine similarity between adjacent-stage topics. The specific steps are as follows:

#### Text preprocessing

We standardized and supplemented the exported bibliographic data and built a custom dictionary and stop-word list. We segmented the abstract text, replaced synonyms, removed stop words, and built a dictionary and corpus according to the bag-of-words model. Among them, the self-defined dictionary was derived from the keywords included in this study: Stanford University stop list [19], Harbin Institute of Technology stop list, Baidu stop list [20], and NLTK stop list [21] were used for the summary of the stop list. The stop list was supplemented according to the pre-segmentation results to remove words with high word frequency but no practical meaning. Chinese and English word segmentations use the Jieba and NLTK modules in Python, respectively, to segment the abstract text based on the custom dictionary in precise mode. Simultaneously, the term frequency-inverse document frequency (TF-IDF) algorithm is used to extract text features, and the importance of each term in the document is quantified to verify keyword relevance in the corpus [22] to improve the accuracy and interpretation of the topic model.

#### LDA topic model

LDA, first proposed by Blei et al. [23], is a probabilistic topic model that identifies hidden semantic structures in unstructured text for topic abstraction and clustering. The model comprises a three-layer Bayesian network structure of “document-topic-word,” where documents include several topics. Each topic comprises a specific set of words from the document, and each word in the document is associated with a certain probability distribution. Thus, the topic of a document can be represented by the group of words with the highest probability of occurrence [24]. Thus, this model obtains the document situation of a specific topic in the DRG domain through “document-topic probability distribution.” It obtains the potential topic and word distribution of different topics in the DRG domain through “topic-word probability distribution,” [25] thereby calculating the semantic relevance between topics and documents and between topics and words. The study utilizes the Gensim library in the Python language to construct a topic model. After debugging and validation, the hyperparameters α and β are set to 0.1 and 0.01, respectively, with 1000 iterations and 5 passes over the corpus. The resulting topic model, under these settings, ensures sufficient training and convergence while possessing good robustness and generalization capabilities.

The LDA topic model must set the number of topics in advance, and the common method is to calculate the topic perplexity and coherence [26]. The degree of confusion [27] measures the quality of a probability distribution or probability prediction sample and determines the optimal number of topics. The smaller the degree of confusion, the more stable the topic structure of the model is and the less topic uncertainty there is. Topic consistency [28] is used to describe the distribution distance between different topics. We used the sliding-window-based coefficient of variation (CV) method to calculate the consistency. The higher the score, the higher the discrimination between topics and the better the clustering effect of the model. We used perplexity and coherence score metrics, along with visual analysis, to select the optimal number of topics, aiming for low perplexity and high coherence scores.

Confusion is calculated as follows:

$$\mathbf{Perplexity}\boldsymbol\;\left(\mathbf D\right)\boldsymbol\;\boldsymbol=\boldsymbol\;\mathbf{exp}\boldsymbol\;\left\{\boldsymbol-\frac{\boldsymbol\sum_{\mathbf d\boldsymbol=\mathbf1}^{\mathbf M}\boldsymbol\;\mathbf{log}\boldsymbol\;\mathbf p\boldsymbol\;\left({\mathbf w}_{\mathbf d}\right)}{\boldsymbol\sum_{\mathbf d\boldsymbol=\mathbf1}^{\mathbf M}\boldsymbol\;{\mathbf N}_{\mathbf d}}\right\}$$

where D is the document set, exp{} is an exponential function with the natural logarithm e as the base, $\mathbf{p}({\mathbf{w}}_{\mathbf{d}})$ is the generation probability of document d, ${{\varvec{N}}}_{{\varvec{d}}}$ represents the lexical length of document d, and M is the number of documents.

The consistency score is calculated as follows:

$$\mathbf C\mathbf o\mathbf h\mathbf e\mathbf r\mathbf e\mathbf n\mathbf c\mathbf e\boldsymbol\;\left(\mathbf V\right)\boldsymbol\;=\sum\limits_{\left({\mathbf v}_{\mathbf i},{\mathbf v}_{\mathbf j}\in\mathbf V\right)}\mathbf s\mathbf c\mathbf o\mathbf r\mathbf e\boldsymbol\;\left({\mathbf v}_{\mathbf i},{\mathbf v}_{\mathbf j},\boldsymbol\in\right)$$

Here, V is a set of words describing the topic, and ${\varvec{\upepsilon}}$ returns a smoothing factor for the real number to guarantee the score.

#### Thematic similarity

Topics in similar developmental stages often exhibit high similarity in the evolutionary process, and the evolutionary relationships between different topics can be identified by extracting topics with high similarity. The cosine similarity is a widely used measure that assesses the similarity between topics by measuring the angle between two vectors, thereby determining the degree of correlation and evolutionary path of topics [29]. The range of cosine values was [0, 1]. The closer the cosine value is to 1, the higher the similarity of the topic vector, and vice versa. Based on existing related research, the average cosine similarity of adjacent temporal topic stages is used as a threshold, where if the cosine value between two topics is greater than the mean, it is considered that there is an evolutionary relationship between the two topics [30]. The cosine similarity is calculated as follows:

$$\mathbf c\mathbf o\mathbf s\mathbf i\mathbf n\mathbf e\mathbf s\mathbf i\mathbf m\mathbf i\mathbf l\mathbf a\mathbf r\mathbf i\mathbf t\mathbf y\boldsymbol\;\mathbf{\left({A,B}\right)}\;=\mathbf c\mathbf o\mathbf s\;\mathbf{\left(\theta\right)}=\frac{\mathbf A\cdot\mathbf B}{\mathbf A\mathbf B}=\frac{\boldsymbol\sum_{\mathbf i\boldsymbol=\mathbf1}^{\mathbf n}{\mathbf A}_{\mathbf i}{\mathbf B}_{\mathbf i}}{\sqrt{\boldsymbol\sum_{\mathbf i\boldsymbol=\mathbf1}^{\mathbf n}\mathbf A_{\mathbf i}^{\mathbf2}}\sqrt{\boldsymbol\sum_{\mathbf i\boldsymbol=\mathbf1}^{\mathbf n}\mathbf B_{\mathbf i}^{\mathbf2}}}$$

Here, Ai and Bi are vector representations of the two topics A and B.

#### Thematic intensity

Topic intensity is the degree of attention paid to a topic in a certain period, and its expression is the number of documents containing the topic. The greater the topic’s intensity, the more likely it is to be considered a hot topic.

The formula for calculating the theme intensity is as follows:

$${{\varvec{T}}}_{{\varvec{k}}}=\frac{\sum_{{\varvec{d}}=1}^{{\varvec{M}}}{{\varvec{\theta}}}_{{\varvec{k}}}^{{\varvec{d}}}}{{\varvec{M}}}$$

Here, ${{\varvec{T}}}_{{\varvec{k}}}$ is the intensity value of the topic k, and ${{\varvec{\theta}}}_{{\varvec{k}}}^{{\varvec{d}}}$ is the probability of the topic k appearing in the document d.

## Results

### Time and geographical distribution

#### Time distribution

**Fig. 1:**
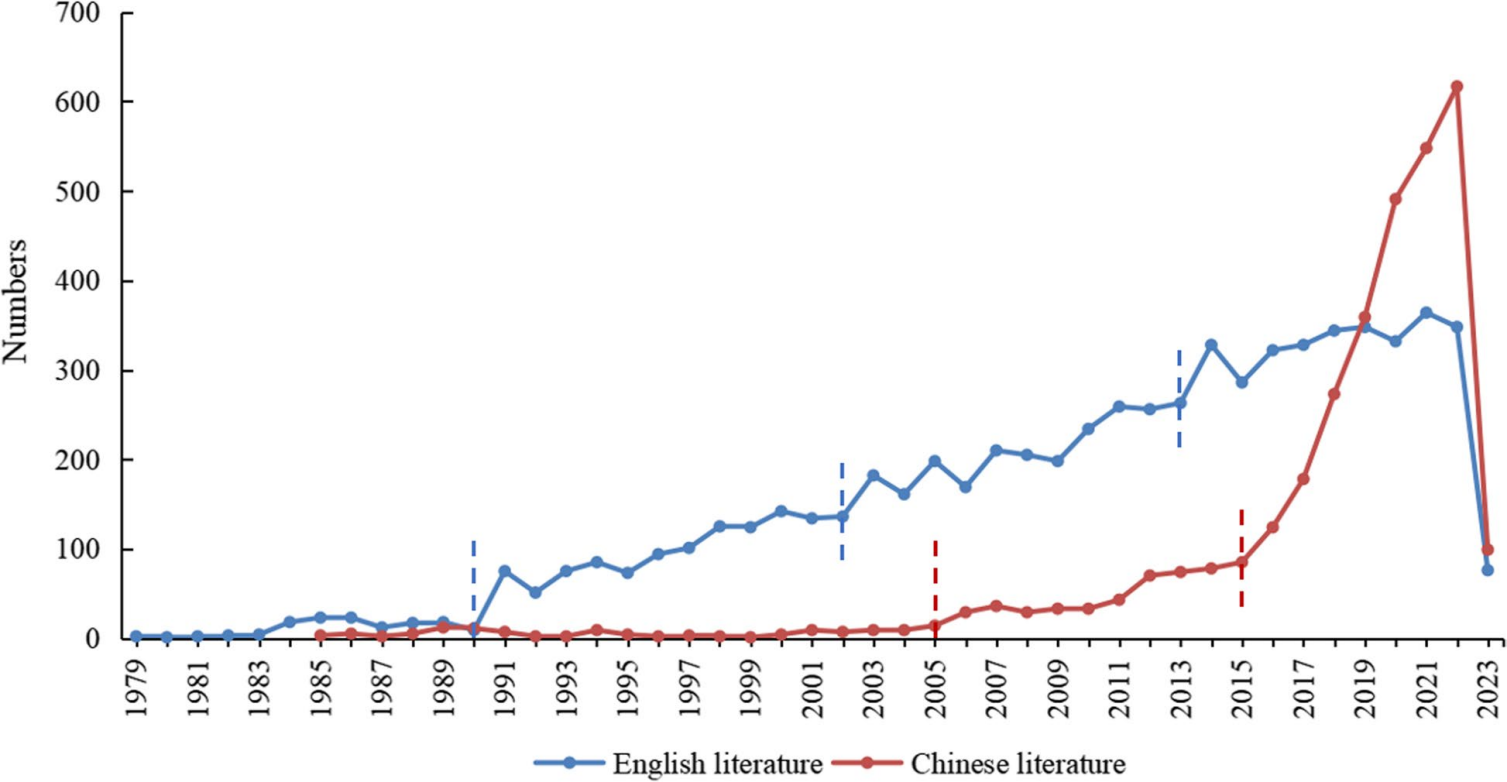
Annual trend of diagnosis-related group (DRG) publications in Chinese and international contexts. Note: The study data search deadline was April 12, 2023. Therefore, the literature data for 2023 were incomplete

Since the late 1970s, DRGs have predominantly been studied abroad. With the implementation of DRGs in an increasing number of countries and regions, the number of related studies is also increasing annually. According to the trend in the literature quantity distribution curve, the development of foreign DRGs can be divided into four stages: exploration stage (1979–1990), embryonic stage (1991–2002), development stage (2003–2013), and maturity stage (2014–2023). The number of published articles has shown a steady growth trend overall, and research in the field of DRGs in developed countries has matured. In the late 1980s, China introduced the concept of DRGs to control unreasonable growth in medical expenses and reduce the medical burden on patients. According to the number of papers published, the development of DRGs in China can be divided into three stages: the initial trial period (1985–2005), the active exploration period (2006–2015), and the rapid development period (2016–2023). Figure 1 shows the trend in the number of annual publications of DRG-related literature, both in China and internationally.

#### Geographical distribution

**Fig. 2:**
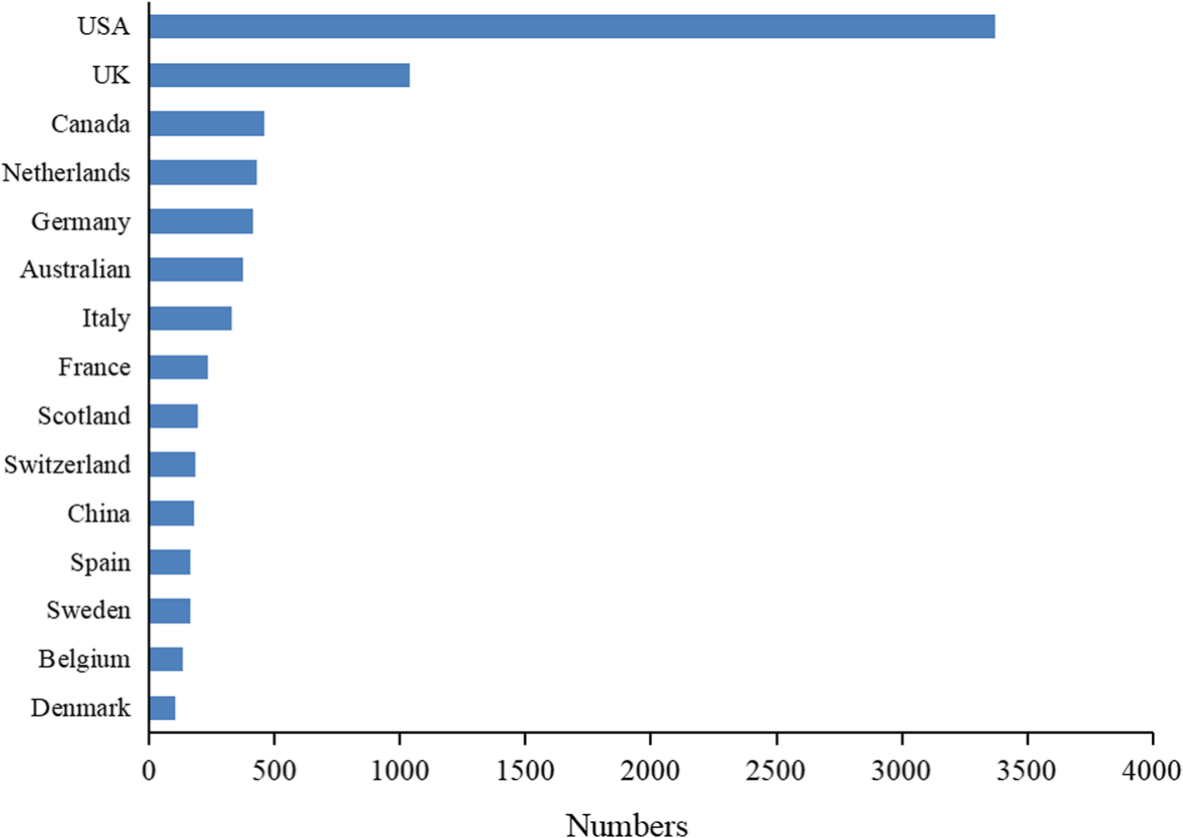
Top 10 countries for DRG-related research and number of publications

Figure 2 shows the number of publications issued by countries worldwide. Research in the field of DRGs has primarily been conducted in developed countries and concentrated in the USA and Europe. The top five countries in terms of the number of publications were the USA, UK, Canada, Netherlands, and Germany. Among them, the USA is the origin of payment according to the disease diagnosis group and takes the lead in applying DRGs to the settlement process of hospitalization expenses, becoming the leading country in the field of DRGs, and its number of publications far exceeds that of other countries. The global proportion of articles published by China is only 2.69%. There is still much room for development in this field, and international influence needs further improvement.

**Fig. 3:**
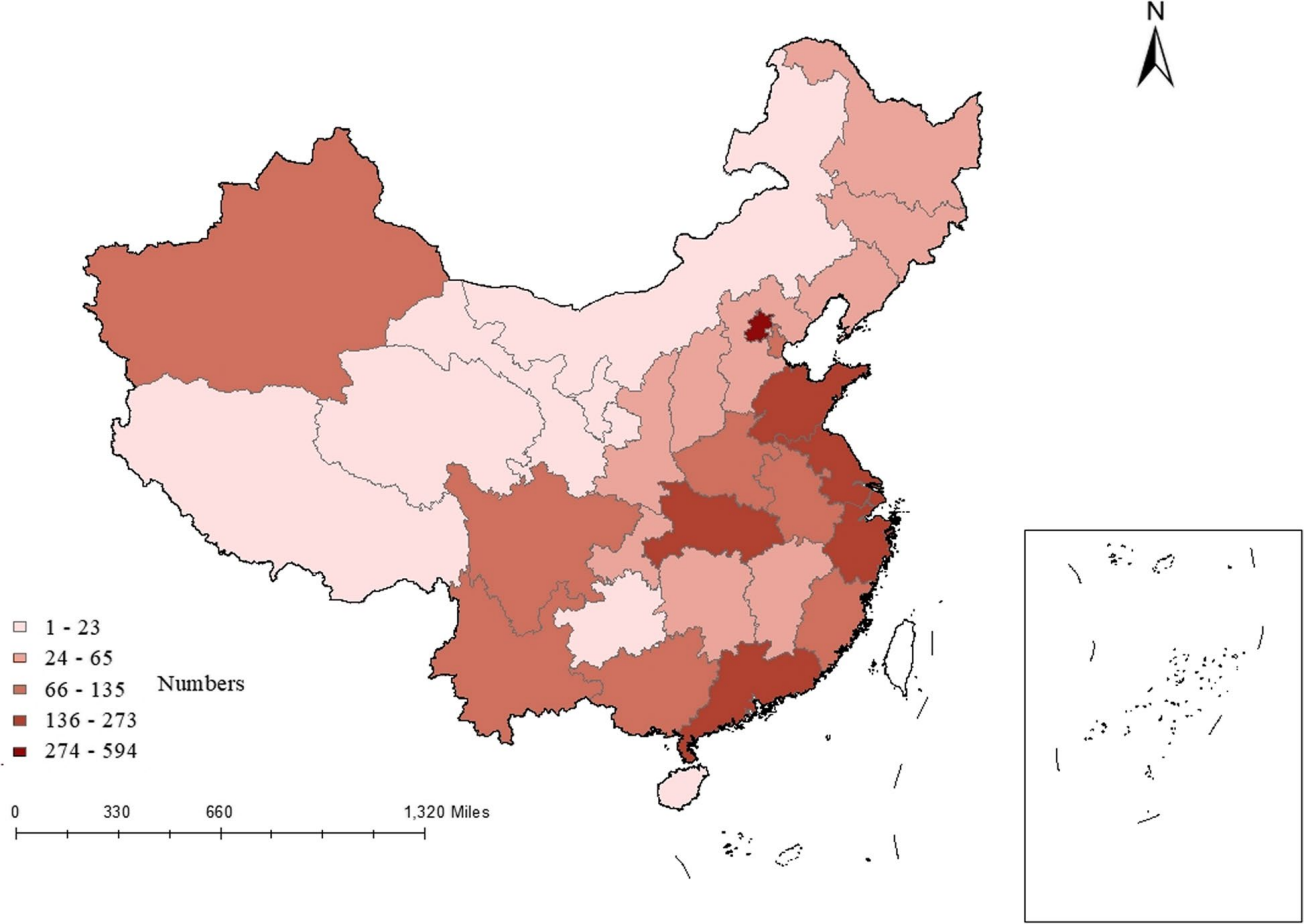
Regional distribution of the number of DRG-related publications in China

Figure 3 shows a visual analysis of the regions to which documents are sent in China. Therefore, research in the field of DRGs is mainly concentrated in eastern coastal areas, such as Beijing, Shanghai, and other economically developed areas. Among these, Beijing, the leading city in the development of DRGs in China, has the largest number of publications. Note that the number of publications issued in Xinjiang is outstanding, and the reform of medical and health systems and high-quality development in western China are effective.

### Topic extraction and analysis based on LDA topic model

#### Determination of the optimal number of topics

**Fig. 4:**
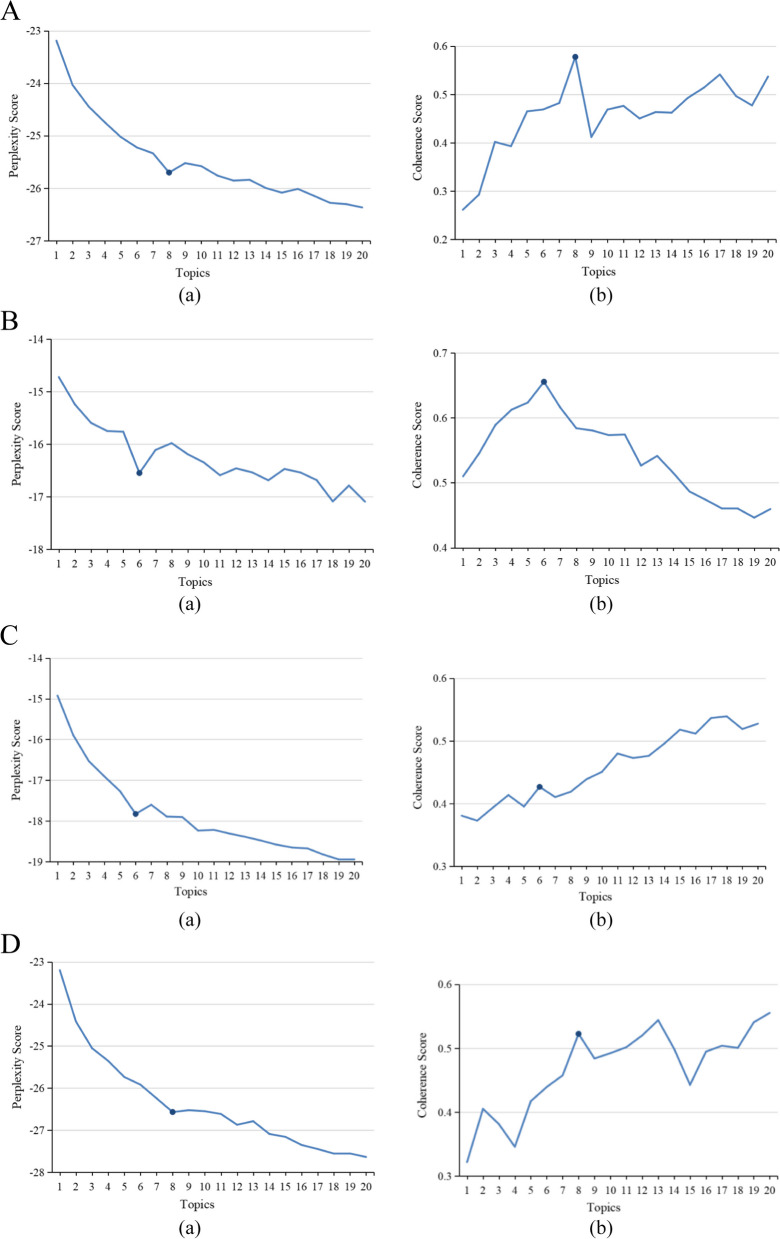
**A** Determination of the optimal number of topics over the entire period. (a) Perplexity Score; (b) Coherence Score. **B** Determination of the optimal number of topics over the initial trial period. (a) Perplexity Score; (b) Coherence Score. **C** Determination of the optimal number of topics over the active exploration period. (a) Perplexity Score; (b) Coherence Score. **D** Determination of the optimal number of topics over the rapid development period. (a) Perplexity Score; (b) Coherence Score

The optimal number of topics was selected by combining the confusion and consistency scores, and the number of topics with smaller confusion and larger consistency scores was selected by combining the visualization results. Taking Chinese literature as an example, we selected an integer within the range of 1 to 20 as the number of potential topics and calculated the corresponding topic confusion and consistency scores, as illustrated in Fig. 4A. When the number of topics K = 8, the degree of confusion is relatively low and the consistency score is relatively high, and both have obvious inflection points. Therefore, the number of topics K = 8 was selected as the optimal number of topics for the entire cycle of Chinese literature. Similarly, according to Fig. 4B, C and D, the optimal numbers of topics for 1985–2005, 2006–2015, and 2016–2023 were determined to be K = 6, K = 6, and K = 8, respectively. Similarly, it can be determined that the optimal number of topics for the full period and each period of the English literature is K = 6, K = 5, K = 8, K = 5, and K = 6, respectively.

#### Topic content analysis

Based on the modeling results of LDA topics, several topics have been studied in the field of DRGs in China and internationally, and the word distribution of each topic can be obtained, namely, “theme-vocabulary probability distribution.” The top 10 featured words with high probability under each topic were sorted and summarized according to the featured words to summarize the topic name and form a topic list in the field of DRGs research.

Among them, the six topics in the English literature were payment systems and cost analysis, effect evaluation and performance management, disease mortality and status assessment, case grouping based on disease severity, internal medicine nursing and quality control, surgical procedures, and operating room management. Table 1 presents the topic summary and subject keywords. The eight topics in the Chinese literature were payment mechanisms, medical expenses, the front sheet of medical records, reform background, performance management, medical service, clinical pathway, and effectiveness evaluation. The topic summary and subject keywords are presented in Table 2.

**Table 1:** Probability distribution of topics and keywords in English literature

Topics	Name	Keywords
1	payment system and cost analysis	DRGs cost charges hospital reimbursement patient medical prospective_payment_system cost-effectiveness analysis
2	effect evaluation and performance management	length_of_stay DRGs hospital performance readmission quality inpatient indicators variation rate
3	disease mortality and status assessment	mortality trauma survival risk SAPS_II death ICU score outcome nosocomial
4	study on case grouping based on disease severity	patient stroke diabetes dialysis delirium age asthma admission severity complication nutrition
5	internal medicine nursing and quality control	hospital physician care resident measure nursing health service efficiency quality
6	surgical procedures and operating room management	ICU blood cholecystectomy surgeon procedure surgery transfusion intensive_care risk complication

**Table 2:** Probability distribution of topics and keywords in Chinese literature

Topics	Name	Keywords
1	payment mechanism	hospitalization_cost case_mix length_of_stay new_rural_cooperative_medical_system DRGs average_length_of_stay medical_cost expense_standard decision_tree statistics
2	medical expense	hospitalization_cost medical_expense Australia medical_institutions payment_methods case_mix insurance single_disease DRGs national_conditions
3	front sheet of medical record	medical_insurance insurance_payment DRGs front_sheet_of_medical_record prepayment_system quality_of_medical_record CMI operation_coding ICD error_rate
4	reform background	medical_expense medical_insurance DRGs high_cost payment_method insurance_fund payment_ reform insurance_system medical_reform expense_control
5	performance management	DRG case_mix public_hospital medical_service pilot_hospital performance hospital_management performance_evaluation medical_staff medical_institution
6	medical service	new_rural_cooperative_medical_system DRGs payment_system single_disease hospitalization_service successful_experience public_hospital medical_institution medical_service RBRVS
7	clinical pathway	public_hospital clinical_pathway DRGs-PPS public_hospital_reform medical_institutions medical_quality cost_accounting overtreatment medical_expense cost_management
8	effectiveness evaluation	Medical_quality medical_expenses hospitalization_expense average_length_of_stay cost_consumption_index CMI time_consumption_index statistics DRGs-PPS public_hospital

### Hot topics analysis

**Fig. 5:**
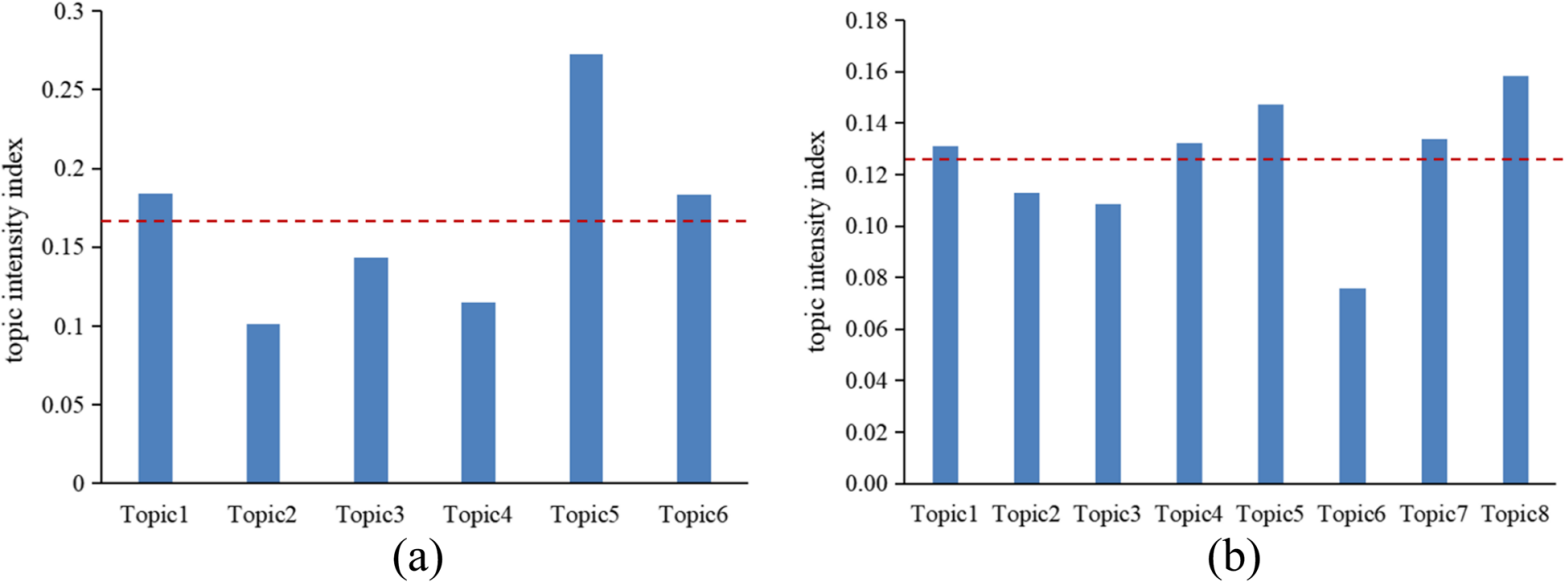
Histogram of topic intensity corresponding to each topic category. **a** English literature; **b** Chinese literature

Based on the LDA topic mining results, the probability of topic k appearing in document d can be obtained, namely “document-topic probability distribution,” which represents the probability distribution of each topic in each document. The larger the probability value, the more likely it is that a document belongs to this topic. The topic intensity corresponding to each topic category is calculated according to the document-topic probability distribution, as shown in Fig. 5. The red dotted line is the average intensity of each topic, and the topic with a topic intensity higher than the average intensity value is a hot topic in the DRG field. The figure shows that the hot topics in the field of DRGs abroad are Topic1 (payment system and cost analysis), Topic5 (internal medicine nursing and quality control), and Topic6 (internal medicine nursing and quality control), while those in China are Topic1 (payment mechanism), Topic4 (reform background), Topic5 (performance management), Topic7 (clinical pathway), and Topic8 (effectiveness evaluation).

### Analysis of theme evolution path

**Fig. 6:**
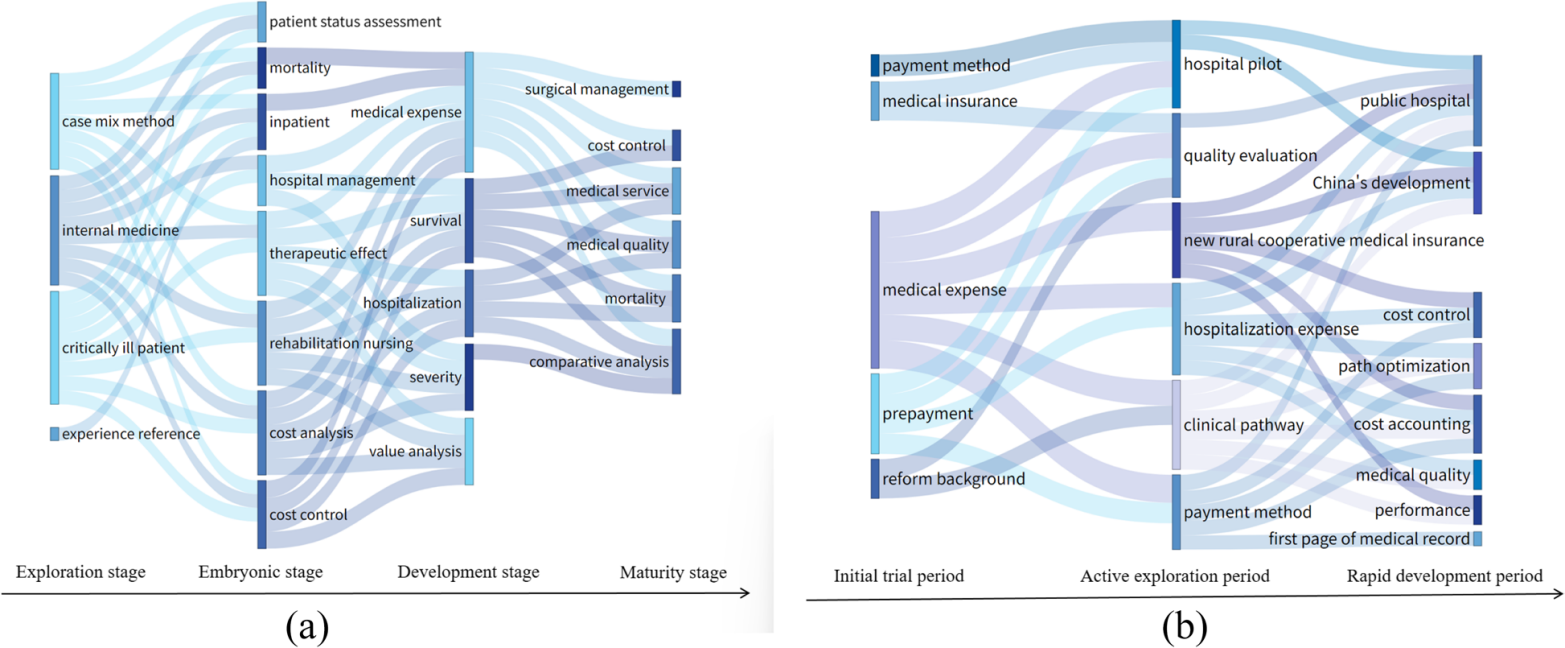
Sankey diagram of topic evolution. **a** English literature; **b** Chinese literature

After LDA topic modeling was performed on the literature at each stage, the cosine similarity between topics in adjacent time stages was calculated. Based on the cosine similarity with the evolutionary relationship, a Sankey diagram of topic evolution was drawn, as shown in Fig. 6. The evolutionary path and logical relationship between topics were obtained. The horizontal axis represents each stage of DRGs’ development in China and internationally; the vertical axis represents topics at different development stages; and the connecting line develops from left to right. The thickness of the line between rectangular blocks represents the similarity of topics. The thicker the line between different topics, the higher the similarity, and the stronger the evolutionary relationship between topics.

## Discussion

The payment system for disease DRGs is a type of homogeneous case-planning based on disease diagnosis and operation. Its practical application controls medical expenses and improves the medical environment. Global research into DRGs continues to increase. After the reform of payment modes in China, the quantity and quality of DRG-related research have increased drastically; however, there is still a gap compared with developed countries. Based on the above results, the following conclusions can be drawn:

### DRG research in China has developed rapidly, but regional development remains uneven

Globally, English literature on DRGs was published earlier than Chinese literature, and the number of published articles generally showed an increasing trend. In the 1970s, Robert B. Fetter and John D. Thompson of Yale University in the USA pioneered the design and development of the payment system for DRGs [31], marking the historic milestone of case-based payment based on clinical and resource consumption similarity. The DRGs payment system entered an “exploration stage.” Since the 1990s, most high-income countries have adopted the DRGs payment method as the primary means of reimbursing hospital acute inpatient expenses. Building upon the USA DRGs, countries have successfully explored reforms adapted to their own DRG systems [32–34], ushering DRG development into an “embryonic stage.” Additionally, Liu [35] has elucidated the sudden surge in literature volume in 1991 based on database constraints. Subsequently, the literature volume has steadily increased, aligning with Liu et al.’s observations [36]. Furthermore, with expanded patient coverage, new medical technologies, and standardized diagnosis and treatment coding, the USA has developed a medical payment system based on an international single-disease grouping system. DRGs have thus been promoted globally as a revolutionary means of medical quality management and cost reimbursement [37], entering a “development stage.” In recent years, the number of studies has stabilized, indicating that research on DRGs in developed countries has reached a “maturity stage” [38]. China began to focus on DRGs in the 1980s and made its first attempt at a large-scale DRGs pilot study in hospitals in Beijing. However, given the relatively backward informatization in China at that time, no unified electronic medical record was established. Additionally, information data could conduct DRG-related research [39]; therefore, the number of DRG studies was low, and this was the “initial trial period.” In 2006, China’s DRG system began to conduct localization development and advocated that regions with conditions could gradually explore the method of payment by disease groups. The advance of network technology, standardization of electronic medical records, and policy formulation have fostered the development of DRGs [40]. The number of DRGs issued showed an initial increasing trend, called an “active exploration period.” In 2016, China began to issue guidance on deepening the reform of basic medical insurance payment methods at the national level, as well as the CHS-DRG version of disease diagnosis-related grouping based on China’s national conditions, and took 30 cities as the national pilot for DRG payment. DRGs began to enter the actual payment stage [41–43], during which the number of DRGs issued in China significantly increased, called a “rapid development period.”

Research on DRGs in China is primarily distributed in the eastern coastal areas, which may be because DRGs were first piloted in Beijing once they were introduced into China. The information technology development in Beijing, Shanghai, Guangdong, and other first-tier cities occurred earlier, and the flow of scientific research institutions and talent was more concentrated. Therefore, more relevant literature existed. In contrast, owing to the relatively backward information level, insufficient supply of medical resources, and uneven distribution of talent in western China [44], the amount of DRG research literature is relatively small. However, compared with other provinces and cities in the west, Xinjiang has a relatively large number of papers, which may be because it covers one-sixth of the total area of China. Its strategic position and geographical advantages are more prominent, making it representative of the western region [45]. Among them, Urumqi City, an early pilot city of national payment according to disease DRGs, performed the actual payment of DRGs in six local hospitals. Therefore, the research effect in Xinjiang requires attention.

### DRGs’ wide range of research topics in China and insufficient research depth

The LDA topic modeling shows that foreign countries also focus on the difficult problems of diseases and fields not covered by DRGs based on the payment policy environment of DRGs. The topics include influencing factors and optimization suggestions for DRG group payment based on the severity of diseases, medical disease nursing, and surgical operations, as well as research on DRGs of nursing home versions, DRGs of home care versions, and DRGs of long-term care [46]. Chinese research has primarily focused on exploring and applying DRG reform background analysis, payment mechanism research, cost accounting, implementation effect evaluation, medical quality management, and other topics. Simultaneously, to continuously strengthen medical quality, standardize medical behavior, and save medical resources during DRG implementation, clinical pathway management, quality control of medical record first page and coding operation, selection of performance appraisal indicators, and other topics have also attracted attention. This may be due to moral hazard behaviors such as high coding problems, patient selection problems, inhibition of new technology and business, and investment reductions in disease prevention and health promotion [10, 47]. Research on DRGs in China is progressing; however, compared with the mature experience abroad, China prefers to study the theoretical framework and practical exploration of DRGs as a whole. China lacks refined research on the DRG implementation path from a disease perspective. Research on quality control in the operation process of DRGs is not mature. Therefore, the overall implementation depth is insufficient, and the research field must be expanded.

Additionally, the subject intensity shows that the research hotspots of foreign scholars in the field of DRGs focus on care quality for specific diseases in surgery, the impact of surgical procedures on DRGs, and cost analysis and payment system research on DRGs. However, research hotspots in China focus on mechanism analysis, hospital management, and effect evaluation. Difficult problems such as accurate case grouping of various diseases, continuous innovation of payment modes, and efficient evaluation of medical services require further studies. Yin Yani [48] confirmed this conclusion.

### Development progress of DRGs and ongoing long-term exploration stage

The literature evolution Sangji diagram shows that, after the short-term methodological research, foreign countries began applying DRGs to medical nursing and surgical operations. DRGs were optimized based on indices such as disease severity and patient survival status, and DRGs were used in hospital management, cost analysis, cost control, efficiency evaluation, and quality management. After a long period of development and innovation, DRG-related research has almost reached maturity. However, the research on DRGs in China has experienced the development path of “basic theory-practical application-quality control,” which is consistent with the results of Liu [49] on the evolutionary history of DRGs in developing countries. Early research focused on basic theory, exploring DRGs in developed countries such as the United States, prepaid mechanisms, and proposing a group payment strategy suitable for China. In the middle stage, most studies were practical explorations and comparative analyses. China has begun to conduct application research on DRGs, focusing on hospital pilot research, medical insurance system reform, payment mode comparisons, and implementation effect evaluations. Among them, the negative impact of implementing DRGs has attracted the attention of many scholars. Therefore, research on quality evaluation and clinical pathways has gradually increased in this stage to strengthen the standardized management of medical diagnosis and treatment processes. In recent years, China has begun to conduct DRG hospital management and quality control-related research, including performance management, medical record first page, and path optimization.

From the evolutionary path of the development of DRGs, DRGs research in China is becoming increasingly improved; however, it remains in the long-term reference and exploration stages. The challenges posed by implementing DRGs to healthcare quality continue to warrant attention. Gu [50] believed that the payment mode of DRGs in China is only in the experimental stage and that the healthcare supply system lacks sufficient governance structure and incentive measures. This indicates that a management system that is more suitable for national conditions in the pilot process of DRGs must be found. Simultaneously, topics related to expenses run through the entire path cycle. Combined with the rapid development of the level of medical services in China, controlling medical expense growth has become the core goal and research focus of DRGs. Li [51] reached a similar conclusion in their review; that is, the main research content of DRGs in China is medical expenses. Thus, Chinese research should analyze the factors influencing medical expenses based on the DRG payment system. The results of the research hotspots and theme evolution paths show that research trends in DRGs will focus on medical quality management, cost control effect evaluation, accurate case grouping, and implementation path optimization.

The above conclusions show that after the pilot and application of the DRGs payment mode in China, research has made breakthroughs. However, compared to its overall promotion and application in developed countries, it faces challenges. Therefore, China should strengthen cooperation between Chinese and international academic institutions, understand the latest research results and practical experience, and actively explore and implement a payment system adapted to its own national conditions. China should further optimize the allocation of medical resources, improve the quality and efficiency of medical services, realize the reform and innovation of medical insurance payment methods, and provide assistance for popularizing and applying DRGs in China. The details are as follows:

- First, the resources of all regions are balanced, and effective coverage of the reform of payment methods is achieved. DRG pilot areas should be extended from the national to the interior level. DRG management mode and reimbursement standards should be formulated in line with actual regional conditions according to the economic conditions and epidemiological characteristics of each region. Provincial reform pilots paid by DRGs should be promoted, and the regional coverage rate of DRG reform should be improved. Simultaneously, multi-party cooperation should be strengthened among government departments and medical institutions at all levels. Special DRG working groups, or expert advisory committees, should be established. Meetings, seminars, and experience-exchange activities should be regularly organized. Information sharing, communication, and DRG research and application should be promoted nationwide.
- Second, departmental linkage is strengthened, and the research depth is improved in the DRG field. Considering the key and difficult problems in implementing DRGs, while continuously expanding the research field, research on implementing DRGs should be refined to ensure the accuracy and applicability of DRGs in different disease fields. Simultaneously, through the coordination and cooperation of health departments, financial departments, medical insurance departments, and other departments, the coordinated promotion of system reform, payment reform, performance reform, and revenue reform forms policy integration and optimal allocation of resources and realizes the deep integration of multiple reform achievements based on the linkage of medical treatment, medical insurance, and medicine.
- Third, theory is combined with practice to promote high-quality medical reform. Scientific, transparent, and operable theoretical frameworks are formulated and improved, such as the DRG classification standard, cost weight calculation method, and clinical pathway management guide; providing operational guidance for the quality control of medical institutions; and promoting fine management and operational efficiency improvement in hospitals. Simultaneously, aiming at the difficulties and problems in the practice process, such as medical data collection, filling in the first page of medical records, and weight calculation rules, we will provide corresponding training, consultation, and technical support to help medical institutions fully understand and reasonably apply the DRGs system and provide an empirical basis and improvement direction for theoretical research. Additionally, a high-quality, strict monitoring and evaluation system should regularly evaluate the impact of DRGs, identify problems, and make timely improvements to inform policy adjustments and enhance DRG application in China’s medical system.

## Conclusions

We analyzed the time and regional distribution of Chinese and international publications by comprehensively searching Chinese and English databases. We used the LDA model to mine potential topics in Chinese and international DRGs. We also summarized the hot topics of DRG research based on the calculation of topic intensity. We analyzed the evolution path and research trend of DRGs development based on the calculation of cosine similarity between topics. It is a reference for in-depth research and future research on DRGs. In DRG development abroad, the research hotspots primarily focused on exploring DRGs, related research under the payment policy environment, and uncovered fields of DRGs. In contrast, the Chinese research started late, primarily focusing on basic theory research and foreign experience and then gradually turning to practical exploration and comparative analysis research. In recent years, Chinese research has focused mainly on hospital management and quality control. In the future, Chinese research should focus on the quality of medical care during the implementation of DRGs and analyze the influencing factors of medical expenses from the perspective of DRGs. Future research should also explore related fields such as cost control effect evaluation, accurate case grouping, and clinical pathway optimization. Additionally, DRG optimization management should be strengthened for complex and high-risk diseases, as well as for special populations. This would promote DRG application and popularization in China. Strengthening academic exchange and cooperation, deepening coordination between medical insurance and relevant departments, and establishing a reform mechanism integrating research and practice will enhance China’s medical quality management, optimize resource allocation, control expenses, and contribute to global healthcare development.

## Limitations

First, the literature sample was only selected from the WoS and CNKI databases. Although these databases are authoritative, several DRGs-related literatures published in other databases may still be potentially omitted. Therefore, future research should include additional databases. Second, regarding language, this study only included literature in Chinese and English. Future research should broaden language selections. Additionally, although the LDA model overcomes the one-sidedness of using journal literature keywords to mine research hotspots, this study only analyzed abstracts in the literature, and the topic tags were named according to the subject words and subjective judgment, lacking the participation of domain experts. Moreover, literature processing was performed manually, potentially leading to incomplete data collection and omissions.

## Data Availability

The datasets and analysis codes used and/or analyzed during the current study are available from the corresponding author on reasonable request.

## Abbreviations

DRGs: Diagnosis-Related Groups
LDA: Latent Dirichlet Allocation
WoS: Web of Science
SCl-EXPANDED: Science Citation Index Expanded
CNKI: China National Knowledge Infrastructure
TF-IDF: Term Frequency–Inverse Document Frequency
CV: Coefficient of Variation
